# The Field Assessment of Quiescent Egg Populations of *Aedes aegypti* and *Aedes albopictus* during the Dry Season in Tapachula, Chiapas, Mexico, and Its Potential Impact on Vector Control Strategies

**DOI:** 10.3390/insects15100798

**Published:** 2024-10-14

**Authors:** José Ignacio Navarro-Kraul, Luis Alberto Cisneros Vázquez, Keila Elizabeth Paiz-Moscoso, Rogelio Danis-Lozano, Jesús A Dávila-Barboza, Beatriz Lopez-Monroy, Rosa María Sánchez-Casas, Marco Antonio Domínguez-Galera, Pedro Christian Mis-Avila, Ildefonso Fernandez-Salas

**Affiliations:** 1 Laboratorio de Entomología Médica, Facultad de Ciencias Biológicas, Universidad Autónoma de Nuevo León, Monterrey 66450, Nuevo León, Mexico; jose.navarrok@uanl.edu.mx (J.I.N.-K.); jdavilab@uanl.edu.mx (J.A.D.-B.); beatriz.lopezmr@uanl.edu.mx (B.L.-M.); 2 Instituto Nacional de Salud Pública, Centro Regional de Investigación en Salud Pública, Cuernavaca 62100, Morelos, Mexico; luis.cisneros@insp.mx (L.A.C.V.);; 3 Veterinaria y Zootecnia, Facultad de Medicina, Universidad Autónoma de Nuevo León, General Escobedo 66050, Nuevo León, Mexico; 4 Tecnológico Nacional de México, Campus Chetumal, Chetumal 77013, Quintana Roo, Mexicopedrochristianmis@gmail.com (P.C.M.-A.)

**Keywords:** neglected, waste management, ovicide, diapause

## Abstract

**Simple Summary:**

The role of mosquito eggs in disease transmission is not well understood, despite reports of virus vertical transmission and their ability to survive dry periods in a dormant stage. Additionally, field evidence shows that new generations of mosquitoes often reappear weeks or months later in certain city areas. This may be because both species follow an “R strategy” reproduction pattern, laying many eggs to ensure survival in a harsh environment. This study was conducted in southern Mexico in 2023 and focused on the egg-laying activity of *Ae. aegypti* and *Ae. albopictus* mosquitoes during the dry inter-epidemic season. Ovitraps were used in the backyards of the houses to identify egg species, and further larval containers were surveyed. Most of the larval habitats were containers smaller than 1 L, commonly not treated as they were considered trash. Even though homeowners volunteer to remove them during government cleaning community-based campaigns, city waste disposal trucks transporting these containers do not take any measures to destroy the tiny eggs. We conclude with the urgent need to develop an egg-killing treatment, which could significantly enhance vector reduction and achieve a total stage strategy within the Integrated Vector Control Programs.

**Abstract:**

Although integrated management and control programs implement intense control measures for adult, pupal, larval, and breeding sites during outbreaks, there is a lack of studies to understand the role of the vector egg stage in disease dynamics. This study aimed to assess the dry season quiescent *Aedes aegypti* and *Aedes albopictus* egg populations in houses and backyards in Tapachula, southern Mexico. Two hundred and fifty ovitraps were placed in 125 homes in the Las Americas neighborhood. A total of 7290 eggs were collected from 211 (84.4%) ovitraps. Only 5667 (77.7%) hatched under insectary water immersion and food supply conditions, with 4031 (71.1%) identified as *Ae. aegypti*, and 1636 (28.8%) as *Ae*. *albopictus*, respectively. The remaining 1623 (22.3%) did not hatch due to Delayed Hatching and/or quiescence tropical stage. Eighty-three larval containers were sampled with desiccated eggs during the dry season; most of them were described as trash waste because larvicides are only used for larger containers of 5–10 L. Evolutionary characteristics for the two species including partial egg hatching, ambient-regulated quiescence, the ability of the embryo to survive for a more extended period intra-seasonally, the egg sticking to inner container walls, demands urgent operational research to achieve successful egg-proof larval container methods.

## 1. Introduction

The number of dengue cases in the Americas increased to 3,578,414 in 2024, with 2888 severe dengue cases and 1039 deaths reported [1]. Due to the lack of effective vaccines providing immunization against four serotypes of viruses and the limited development of pharmaceutical products, vector control is crucial in the fight to prevent disease outbreaks in these countries. Despite efforts to control the *Aedes aegypti* mosquito, which carries the dengue virus, the measures have only provided short-term results, leading to increased new generations of mosquitoes and correlated dengue cases in some local neighborhoods. Abundant populations of *Ae aegypti* facilitated the emerging and dispersion of new arboviruses from 2013 to 2015 in the Americas region, such as Chikungunya and Zika [2], and, to a lower extent, the invasive *Aedes albopictus* [3]. On the other hand, most government vector control strategies, namely chemical, biological, and educational, focus on the adult, pupal, and larval stages. Control of the egg stage is almost nonexistent; its tiny size, black color, and attachment to the inner sides of artificial containers protect it from the field technicians’ sight and removal. During egg-laying, the female places them individually in a humid film above the water’s surface, which is made by capillarity. It takes about 72 h for whole embryo maturation and egg myelinization. If water evaporation occurs in the following days, this embryo can survive from up to 6 months to two years. Despite its importance during arbovirus transmission, egg populations in *Aedes* mosquitoes have been poorly studied. Nature provided a sticky substance produced by the female accessory gland containing hyaluronic acid to glue her eggs onto the larval site walls [4]. This trait accelerated the vector geographical dispersion mainly through glued eggs in the used tires [5]. Similarly, Lozano-Denis et al. [6] reported that around 5% of the dengue virus in eggs was collected in 20,000 ovitraps in four states in southern Mexico. This high rate of vector transovarial transmission is strong evidence of the epidemiological importance of explaining the outbreaks beginning in endemic places. The question of how these eggs may tolerate desiccation for such a prolonged period relies on a physiological stage defined as diapause. For insects in the tropics, specific types of diapause occur. Diapause and quiescence are two forms of dormancy found in insects. Diapause is characterized as a dormant state that is hormonally programmed in advance and is not immediately ended when favorable conditions are present. On the other hand, quiescence is a dormancy state that occurs in response to unfavorable environmental conditions and ends immediately when favorable conditions return. For instance, the inability of *Ae. aegypti* eggs laid above the water line to hatch are considered quiescent because development can promptly resume upon immersion in water [7].

This study aimed to generate new data on the populations of dormant *Ae. aegypti* and *Ae. albopictus* eggs during the dry season or inter-epidemic period. This study focused on three objectives: (1) using ovitraps to identify the species of female mosquitoes laying eggs, (2) assessing the populations of desiccated eggs and their backyard containers, and (3) evaluating the killing effectiveness of liquid Temephos 5% dissolved in mineral oil as potential ovicide of quiescent eggs.

## 2. Materials and Methods

### 2.1. Study Area

**Figure 1:**
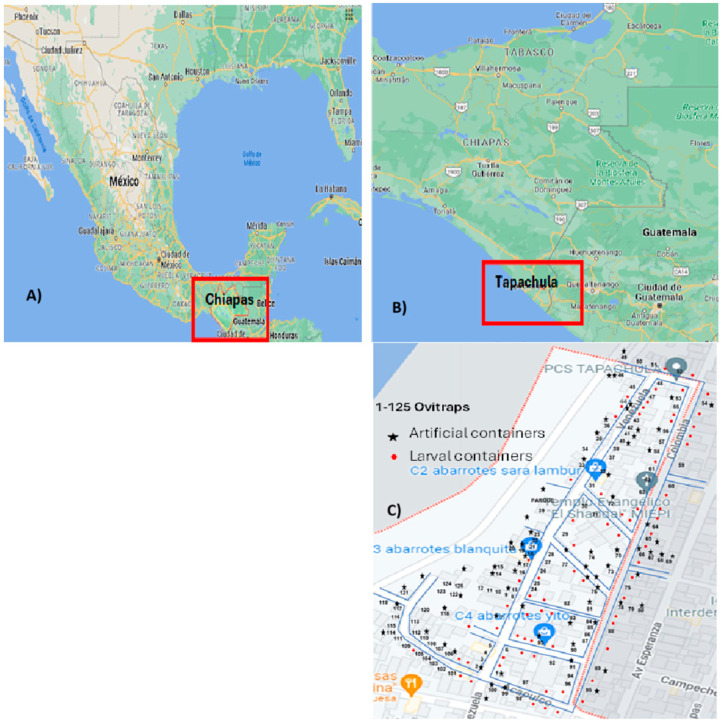
(**A**) Geographic location of the state of Chiapas in the Mexican national territory. (**B**) Locatable 14.9277, longitude: −92.2602. (**C**) Geographic limitations of the Las Americas section II and its border with the Coatán River (Google Maps, @ INEGI 2023).

The research was conducted in the Las Americas section II neighborhood in Tapachula, Chiapas, Mexico. This neighborhood is located 5.13 km east of Tapachula’s geographic center. It is also 3.69 km north of Tapachula’s urban center (Figure 1). The neighborhood covers an area of 11 hectares and includes infrastructure for 309 homes. The population density is 1042 people per square kilometer, with residents of various nationalities [8].

The study area is experiencing disorganized urbanization. It is located next to the Coatán River, which creates ideal shade, humidity, and temperature conditions for *Aedes* mosquitoes. Despite having operational electricity, drainage, and drinking water services, water supply outages occur throughout the year, leading households to use various containers to store water.

**Figure 2:**
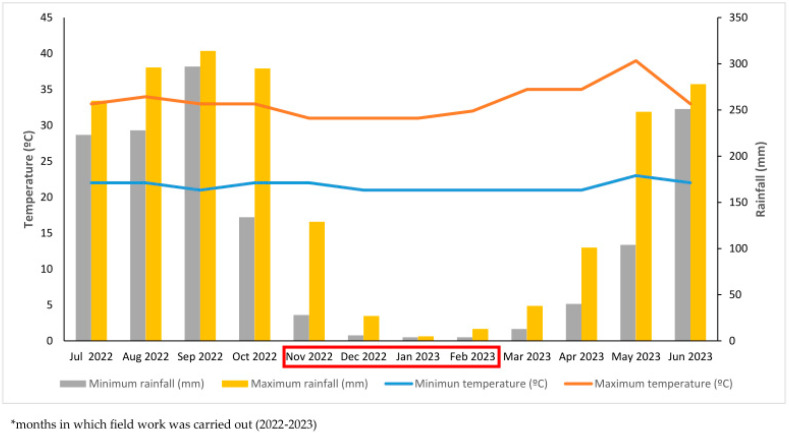
Distribution of rainfall (mm) and temperatures (°C) in Tapachula, Chiapas, Mexico, from July 2022 to July 2023. Red box represents the months in which field work was carried out (2022–2023).

Regarding the local weather patterns, a six-month rainy season is present from June to November, whereas a dry season continues from December to late May in the following year. Annual accumulated water in mm ranges from 1200 to 4000 mm, including coastal areas and a mountain range of 1500 m elevation [9] (Figure 2). Dengue cases are endemic yearly, with a higher monthly incidence during the peak of rainy season months [10].

### 2.2. Identifying In Situ Female Egg-Laying Mosquito Species in Houses Indoors and Outdoors Using Ovitraps

Before beginning home visits, a meeting was held with neighborhood leaders to obtain appropriate authorization. Permission was also requested from the heads of the families to enter the homes, and individual talks were held to explain the project’s objectives and process. Once authorized by the community leader, we presented ourselves duly identified at each home, where we offered a brief presentation about our work and its objectives and answered the residents’ questions.

Monitoring took place from November 2022 to March 2023. Out of 309 houses in the area, 125 (40.45%) agreed to take part (see Figure 1C). Each home was georeferenced, and an intra- and peri-home inspection was conducted to identify larval containers. The inspection also checked if any water containers were treated with larval control methods for *Ae. aegypti* (such as Temephos-abate^®^ or Spinosad^®^) authorized by the state Secretary of Health. Additionally, the residents were interviewed about the latest visit from the Health Secretary’s staff and whether they had used any *Ae. aegypti* control.

Two ovitraps were installed in each home, consisting of a 0.5 L black plastic container filled with 300 mL of tap water and an 8 width × 15 length cm strip of filter paper. One ovitrap was placed in the bedroom, and the other was near water storage sources. They remained there for four days (96 h). On the fifth day, the homes were again visited to collect the egg-positive ovitraps labeled and stored in plastic bags. These samples were taken to the insectary of the Regional Public Health Center of Mexico (CRISP-INSP) in Tapachula. As a first step to stimulate egg hatching, the filter paper strips of each ovitrap were placed in a 1 L plastic beaker containing 750 mL of tap water and larval food. After 24 h and once hatched, the larvae were transferred to individual 22 × 35 × 5 cm trays containing 1.2 L of water and 1.5 cm deep. The first and second instar larvae were fed during the first three days with 0.4 g per tray of a larval diet (mixture of proteins, fats, fiber, and minerals, previously ground, sieved, and sterilized; 5001 Laboratory Rodent Diet (Lab Diet, CA, USA)). The third and fourth instar larvae were fed with 0.8 g per tray of this same diet until the sixth day. Similarly, pupae were transferred to 20 cm-diameter trays and covered with a tricot mesh with rubber bands to confine the adults after their emergence. Adult mosquitoes were counted, sexed, and morphologically identified [11]. On the other hand, the negative ovitraps were washed with tap water, the filter paper strips were replaced, and 300 mL of tap water was added. These ovitraps were placed back at the monitoring site for four additional days, repeating the abovementioned process.

### 2.3. Sampling of Aedes Quiescent Eggs

**Figure 3:**
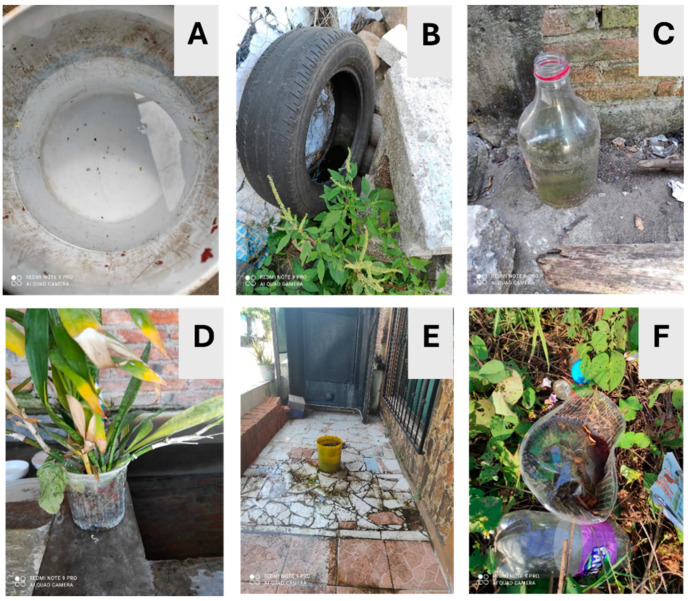
Examples of dry-season breeding sites (wet and dry) identified and monitored for the development of *Aedes* mosquitoes in the Las Americas section II in Tapachula, Chiapas, Mexico. (**A**) A 5 L plastic container with water, (**B**) tire with water, (**C**) 2 L plastic bottle with water, (**D**) plastic vase with plants, (**E**) 20 L plastic container with water, and (**F**) plastic container 5 L with water.

Monitoring took place from January to February 2023. Of the 125 homes that participated in the ovitrap study to detect the presence of *Aedes* mosquitoes, all were authorized to continue with the project’s next phase. As a first step, an inspection was carried out both inside and around the homes to identify possible mosquito breeding sites. All containers, fully or partly filled with water, were examined and visually inspected for eggs and larvae. In contrast, dry containers with favorable conditions to become breeding sites were identified, where only eggs were sought (Figure 3).

All possible 83 dry season breeding sites identified were correctly labeled and geo-referenced. The family heads authorized the physical collection of the breeding sites, which were packed with plastic and taken to the CRISP-INSP laboratory. There, tap water and larval food were added to all sampled containers to stimulate the hatching of the eggs. These first instar larvae obtained from each dry-season breeding site and larvae transported from homes were transferred to 22 length × 35 width × 5 height cm plastic trays containing 1.2 L of water and 1.5 cm deep—the same larval diet and handling until adult emergence were similarly managed like with the ovitrap eggs.

In cases where permission was not obtained to remove breeding sites from households, we collected larvae and added tap water and food as needed. After 48 h, we returned to collect the larvae, which were transported to the CRISP-INSP laboratory for analysis in 4 1/2 × 9″ Whirl-Pak^®^ sample bags.

### 2.4. Temephos 5% Dissolved in Odorless Mineral Spirit Bioassay as Potential Operational Control for Quiescent Egg Populations

Temephos 5% was selected to run bioassays considering the following reasoning: (a) Temephos 1% sand granules are a close organophosphate formulation and well-known larvicide for field operators elsewhere; (b) extreme conditions of elevated temperatures and poor relative humidity during the dry season demand a more challenging product such as the mineral oil with a boiling point of 360 °C along with carrying a higher concentration of 5% of Temephos. Although frequent reports of insecticide resistance are available, the World Health Organization experts keep their recommendation of use [12]. In this experiment, gravid female mosquitoes raised from eggs collected in the study area were released into 30 length × 30 width cm metal cages. Inside each cage, a plastic container with a damp Whatman #2 filter paper was provided for the females to lay their eggs. The eggs on the filter paper were allowed to embryonate for 72 h. Afterward, the developed eggs were counted and randomly distributed into 24 plastic containers, each capable of holding 500 mL, with each container containing between 12 and 65 eggs. Subsequently, the containers were randomly selected, and three different treatments were applied: (a) 3 mL of a 5% Temephos solution (Alabaster 500 EC^®^ + mineral oil), (b) 3 mL of mineral oil, and (c) control treatment where nothing was applied to the eggs. These treatments were applied using a 300 mL capacity hand-held plastic sprayer. Two days after the treatments were applied, 400 mL of clean water and fish food were added to encourage hatching. Monitoring continued for 72 h, and the percentage of eggs hatched in each container was recorded using the following formula: (number of hatched larvae/total number of eggs) × 100. Eight observations were made per treatment, and the bioassay was repeated three times.

### 2.5. Data Analysis

Descriptive statistical and plot distribution analyses were conducted on mosquito *Aedes* hatching, emergence percentages, and proportions of *Ae. aegypti* and *Ae. albopictus* from ovitraps. Likewise, the frequency distribution of the desiccated or quiescent egg types of containers found in the locality was assessed. The independence test of the Mann–Whitney U test for nonparametric data was calculated to compare species hatching rates [13]. The effectiveness of Temephos, 5% as a killing compound on desiccated eggs, was measured by comparing the hatching percentage of each treatment using the Kruskal–Wallis test and the Dwass–Steel–Critchlow–Fligner pairwise comparison.

## 3. Results

### 3.1. Species of Aedes Identified in Adult Stages from Eggs Collected in the Household Ovitraps

**Figure 4:**
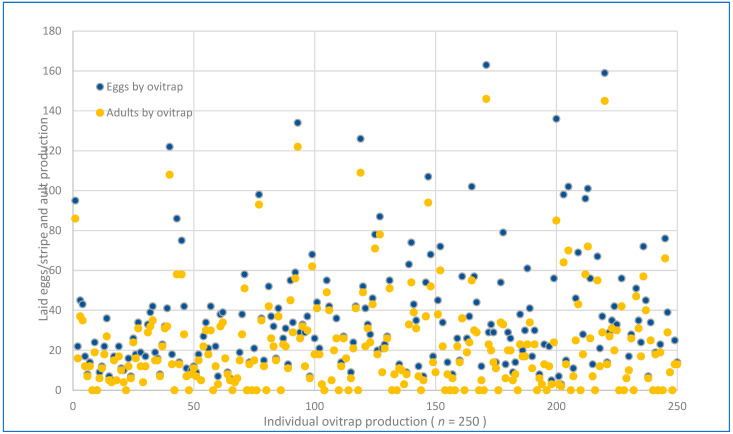
Individual production of 250 ovitraps placed in 125 houses. A total of 7290 eggs were collected in the paper stripes, but only 5667 hatched. Only 40,131 were identified as adult *Aedes aegypti*.

**Figure 5:**
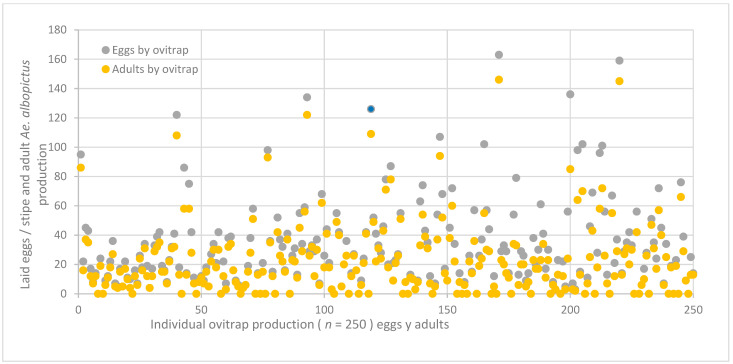
Individual production of 250 ovitraps placed in 125 houses. A total of 7290 eggs were collected in the paper strips, but only 5667 were hatched. Only 1636 were identified as adult *Aedes albopictus*.

Despite the rainy season ending nearly a month prior at the study site, a high level of egg-laying activity was observed. Out of the 250 ovitraps that were placed across 125 participating houses, 211 (84.4%) were found to be positive for *Aedes* eggs, while only 39 (15.6%) tested negative (see Figure 1C). In total, 7290 eggs were recorded, with a pooled hatching rate of 78.5 ± 15.6% (5667 eggs developed into adult stages). Among the adults identified, the majority, accounting for 4031 individuals (71.1%), were *Ae. aegypti,* while 1636 (28.8%) were *Ae. albopictus*. The total count of mosquitoes per species and sex was *Ae. aegypti* 1861 (32.8%) females and 2170 (38.3%) males, and *Ae. albopictus* 767 (13.5%) females and 869 (15.3%) males were found, respectively. Interestingly, the hatching rates per mosquito species were 55.5% for *Ae. aegypti* and 22.4% for *Ae. albopictus*; the null hypothesis stating that the distribution of the groups was rejected. (Mann–Whitney U = 16,878, *p* ≤ 0.005). Eggs of the single species *Ae. aegypti* were found in 209 (83.6%) ovitraps, while only 114 (45.4%) were found for *Ae. albopictus,* respectively. Both mosquitoes were overlapped in 108 ovitraps (43.2%), indicating species co-habitation. On the other hand, 14% of the ovitraps did not attract egg-laying females of the two species. Most of the sampled egg numbers in ovitraps were ≤20 and had a similar distribution pattern in both species. However, some ovitraps recorded ≥100 eggs (Figure 4 and Figure 5).

### 3.2. Sampling and Species Identification of Aedes Quiescent Eggs and Household Wet and Dry Larval Breeding Sites

**Figure 6:**
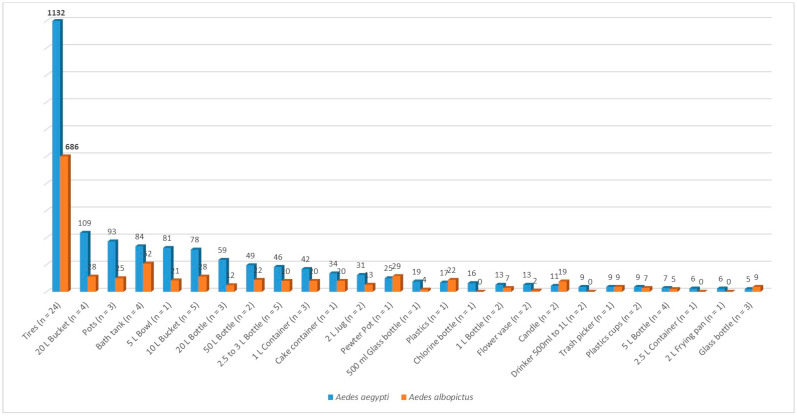
Adult *Aedes aegypti* and *Aedes albopictus* emerging from dry and glued eggs collected above the water line of assorted backyard containers in Tapachula, Chiapas, southern Mexico.

The identification of 500 potential breeding sites for developing *Ae. aegypti* and *Ae. albopictus* mosquitoes was conducted across 125 houses in the Las Americas neighborhood. Only those that could be handled and transported with authorization from the heads of the family were selected. These containers were classified based on their water storage capacity (tires less than 10 L and greater than 10 L). In our sampling, water storage tanks larger than 500 L were not considered since they had been previously treated with the larvicide Spinosad^®^ as part of the Ministry of Health’s control measures. Dry-season larval sites such as tires, 20 L buckets, 3 L vases, 3 L bottles, toilet bowls, 1 L plastic containers, 2 L drinkers, flowerpots, 5 L pots, etc., were collected. In total, 83 potential breeding sites were transported to further monitor the development of larvae and adults. Among the 83 containers examined, 28.9% were discarded tires, while the remaining 71.1% held various types of containers, with 56.6% being smaller than 10 L and 14.5% being larger than 10 L. Within the 77 containers in the community homes collected with water, mosquito eggs were monitored until their adult emergence in the laboratory (Figure 6). A total of 3063 mosquitoes emerged, with 1818 (59.3%) found in tires, 919 (30.0%) in containers with a capacity of less than 20 L, and 326 (10.6%) in containers with a capacity of 20 to 50 L. Of these, 2108 (65.1%) were identified as *Ae. aegypti*, comprising 52.8% females and 47.2% males. Additionally, 1062 (34.5%) were identified as *Ae. albopictus*, with 58.5% male and 41.5% female.

Our study found that a specific set of containers, including three discarded tires, one unused toilet tank, a 2 L cooking pan, and a 1 L plastic bottle, effectively showcased mosquitoes’ quiescent eggs. These containers were collected from household backyards and brought to the research institute. After adding water and larval food to stimulate hatching, we observed the emergence of 188 *Aedes* mosquitoes within 24–48 h under insectary conditions. Of these, 113 (60.1%) were *Ae. aegypti* and 75 (39.9%) were *Ae. albopictus* (Table 1). Frequency distribution of the average eggs and adult production showed discarded tires as the highest larval growing site, even with both species overlapping in southern Mexico. However, more than 50% of available wet and dry containers were also occupied by *Ae. albopictus*, an invasive mosquito with remarkable physiological plasticity (Figure 6).

### 3.3. Effectiveness of Temephos 5% Bioassays on Dried Ae. aegypti Eggs

**Figure 7:**
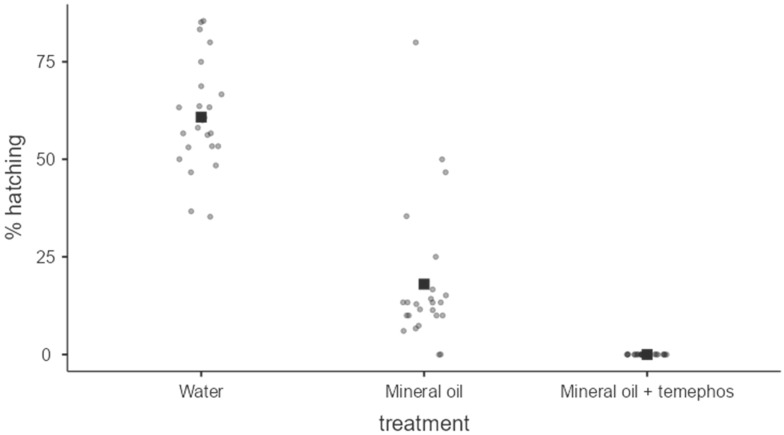
Average percentage (darkest point) of hatching of *Ae. aegypti* eggs exposed to different treatments. Kruskal–Wallis test *X*^2^ = 60.4; fd = 2; *p* < 0.001. Differences in all Dwass–Steel–Critchlow–Fligner pairwise comparisons (*p* < 0.001).

The hatching of dry eggs was fully inhibited by the formulation of 5% Temephos dissolved in mineral spirit oil. This consistency of 100% ovicidal activity was repeated during the three essays, each with eight repetitions (Kruskal–Wallis test *X*^2^ = 60.4; fd = 2; *p* < 0.001, and differences in all Dwass–Steel–Critchlow–Fligner pairwise comparisons (*p* < 0.001). The treatment using only mineral oil resulted in an average of 18% blockage of egg hatching across multiple bioassays and repetitions. The untreated control group had an average hatching rate of 60.8% (Figure 7). Our preliminary results are evidence of the poorly documented ovicidal activity of Temephos for dengue vectors elsewhere. The results also show a potential strategy to apply control measures during the dry inter-epidemic season on small-sized breeding sites carrying quiescent eggs in house backyards and most related sites.

## 4. Discussion

In most dengue-endemic countries in Latin America, routine vector control operations are conducted following WHO Guidelines [14]. However, despite experts’ recommendations, integrated vector control and management strategies are only partially accomplished. Adult mosquitoes are intensively treated using either vehicle-mounted or hand-held space-spraying chemicals. Concurrently, larvae populations are intoxicated in their artificial habitats, mostly with Temephos 1%, Pyriproxyfen, or Spinosad. Although WHO guidelines [14] prioritize environmental management to eliminate larval household containers, which is a very effective action in the field, the truth is that house owners remove only a few larger containers, such as old mattresses and discarded tires. Regarding removing smaller containers, they volunteer to participate in community cleaning campaigns headed by the city authorities. Very often, this task has no supervision, and under dwellers’ judgment, many containers will be beneficial for the future. In other words, only more significant ≥20 L water deposits are treated by government programs, and consequently, thousands of small artificial containers remain in the houses’ backyards. An all-stages *Aedes* control strategy is not being implemented in practice. Whereas the adult, pupae, and larvae stages have some recommended treatments, the lack of mosquito egg-specific control methods is remarkable. Some reasons for the overlooking situation are attributed to the small size of mosquito eggs, ≤1 mm, which are very difficult to see by the busy program technicians; additionally, the eggs are glued to the container’s inner walls with a sticky hyaluronic acid produced by the female accessory gland. Amazingly, after embryogenesis and melanization, the resilient egg populations may resist desiccation for up to 6 months or one year [4].

### 4.1. Aedes Species Laying an Egg in Ovitrap in Early Dry Season

This study, conducted in the early months of the local dry season and linked to the tropical diapause period, found significant quiescent eggs. Of 250 ovitraps settled in 125 participating households, 84.4% showed positive filter paper strips. These eggs developed adults reared and identified as *Ae. aegypti* and *Ae. albopictus*. We observed that both species shared 108 ovitraps (43.2%), which might represent some extent of niche competition of *Ae. albopictus*, an invasive species in Mexico since 1997 [3]. However, the hatching rates of *Ae. albopictus* were significantly lower than *Ae. aegypti*, 22.4%, and 55,5%, respectively. Based on the hatching rates, the egg populations of *Ae. aegypti* in southern Mexico show a form of quiescent dormancy. This is an adaptive response to unfavorable conditions. However, this dormancy can be reversed when dormant eggs are exposed to external stimuli, such as rain. [7]. Conversely, the low result for *Ae. albopictus* may define the need to extend a longer field period associated with Delayed Hatching (DH) overwintering species, a genetically programmed diapause such as in temperate countries [15]. On the other hand, besides the egg stage, the results of significant surviving egg-laying females in the dry period must also be addressed by the control MoH programs. In addition to sporadic scarce and short rains filling temporary larval containers to help females’ survivorship, Delayed Hatching (DH) egg-laying behavior has been reported for some *Aedes* and other mosquito species [16]. Whereas Rapid Hatching species (RH) are used to laying eggs after a 2–3-day embryonic period, DH females may take months or years. The eggs of DH usually hatch at different times, even if they are from the same female. This staggered hatching helps prevent all the larvae from emerging at once in a small space where there is not enough food. Mixed RH and DH behavior is also reported for some species and geographical locations. Both *Aedes* species in southern Mexico probably showed a similar pattern.

### 4.2. Assessment of Quiescent Eggs in Dry Season Collected in Samples Household Containers

As seen in Figure 6, the two groups of larval containers searched for quiescent eggs produced by adult culicines of the two *Aedes* species. The first group consisted of 77 containers, which still maintained some rainwater left from the late rainy and sporadic months during the ongoing dry season. The second group comprised only six containers without rainwater or that were thoroughly dried and screened to look for quiescent and desiccated eggs. Quiescent egg-producing adults of the two vector species were consistently identified in these 77 temporary containers, i.e., *Ae. aegypti in* 100% and *Ae. albopictus* only colonizing 72.0%, respectively. Males and females were almost evenly sampled over ≥95% of 29 assorted categories of larval containers. A total of 3063 males and females of both *Aedes* emerged and were identified, corresponding to 65.4% *Ae. aegypti*, and 34.6% *Ae. albopictus*, respectively. Used tires from the backyards accounted for the most significant adult production after pooling the two species, recording 59.3%. The remaining 2–20 L containers accounted for around 25% more mosquito productivity. Regarding a preferred egg-laying container, both *Aedes* species were found in similar artificial containers in southern Mexico. We assumed that the average higher adult productivity might be attributed to ≥5 L containers sustaining the early dry season vector production due to prolonged water evaporation (Figure 6). From the vector control point of view, we call attention to the smaller “trash-like” containers. They are described as domestic solid waste made of plastics, glass, and paper, which generally may store rainwater and become a larval site. According to Day’s [15] definition, quiescent eggs are dormant but quickly respond to environmental factors such as rainfall; in our case, water and larval food were added, and successful hatching rates were recorded. Therefore, we report field evidence here that they are genuinely quiescent eggs being carried in backyard-specific containers. This finding supports the importance of applying effective measures and not only treating these containers as domestic trash. This result consistently happens since larvicidal activity by government programs is targeted at only treating larger containers ≥5, 10, or 20 L; 55 Ga barrels; 200 L cement tanks; cisterns; etc. The six fully dry containers shown in Table 1 represent findings describing quiescent eggs. Both species were found, accounting for 188 males and females. Similarly, the highest mosquito habitats were used tires, a 2 L cooking pan, and an abandoned toilet tank.

Over the years, we have observed in several cities in Mexico that dengue fever outbreaks are repeatedly reported yearly in the same city hotspots and clusters. Even when the documented log of local program vector control measures describes intense ULV space-spraying, larvicidal activities, and community-based cleaning campaigns with significant clinical case reduction, the rest of Latin American countries also know this in situ epidemic pattern. A likely explanation for these vector population reinfestations when the transmission season begins, causing outbreaks in the same hotspots, is correlated to the neglected quiescent egg populations. We should recall that most insect pests follow the “R” reproductive strategy; they have a higher intrinsic reproductive rate and tend to produce large amounts of eggs and offspring [16]. On the other hand, the impact of control activities between life cycle and disease is widely documented for adult, pupal, and larvae populations. The absence or lack of research on the role of the egg stage is always neglected. The paramount epidemiological importance of vertical virus transmission is firmly documented in many countries. In Mexico, Danis-Lozano et al. [6] report that up to 7.8% of adults collected eggs in ovitraps in nine cities of southern Mexico. Transovarial transmission in infected males is relevant from an entomo–epidemiological point of view since it shortens the transmission cycles in dengue-endemic areas. In this study, a high proportion of males emerging from ovitraps can be considered a risk factor since the probability of infecting wild females is high. Quiescent or uncontrolled *Aedes* eggs can potentially play a role in pathogen maintenance during inter-epidemic periods. Secondly, the ability of the female to glue the eggs in the container’s inner wall using a sticky hyaluronic acid substance has expanded both vector’s geographical distribution. The commercial trade of recycled truck tires in regions and countries better exemplifies this phenomenon [17]. Third, adaptation uses two egg-hatching strategies by *Ae. aegypti* and *Ae. albopictus*, such as quiescent eggs that promptly are stimulated if rain is present: Rapid Hatching (RH) and remaining as a desiccated egg through a Delayed Hatching (DH), patiently waiting for the rainy and outbreak season [1].

### 4.3. Bioassay Temephos 5% as Ovicide

Regarding *Aedes* vector control by government programs, measures are drastically reduced during the dry season. However, many untreated, desiccated, quiescent eggs remain hidden and glued to thousands of small, trash-like containers in residential and non-residential larval habitats [18]. Our results using Temephos 5% dissolved in mineral oil resulted in 100% blocking *Ae. aegypti* egg hatching. We cannot define Temephos as ovicide; perhaps some eggs hatched, and Temephos’s active ingredient intoxicated the first instar stage, acting as a larvicide. However, for practical purposes, it may be a potential tool to eliminate the latent embryo population living in the dry season egg populations. On the other hand, mineral oil, being a high ambient temperature solvent, will keep the active ingredient for 4–6 dry season months after being sprayed in many small backyard containers. Integrated vector control programs may consider including a dry season component against quiescent egg populations [19].

## 5. Conclusions

Although WHO guidelines for vector control during dengue fever outbreaks indicate integrated control measures, the focus is on targeting adult, pupal, and larval stages, with little attention given to controlling the eggs of the *Aedes* species responsible. Frequent observations of city spot epidemics in year-to-year cycles call attention to the possibility of mosquito reinfestations in situ by neglected and uncontrolled dormant eggs. Our study was conducted in Tapachula, a dengue-endemic city in southern Mexico, aiming to generate new insights into the role of eggs in improving vector control in the future. During the dry season months, a group of 250 ovitraps were placed in 125 houses backyards, recording 211 (84.4%) positive ovitraps. Adults who emerged from these eggs were identified as *Ae. aegypti* and *Ae. albopictus*, the two more critical vector species in Mexico. We demonstrated the definition of the quiescent diapause stage because desiccated field-collected eggs responded to external stimuli such as added water and larval food to estimate hatching. Assorted 1–20 L artificial “trash-like” containers were recorded as dry-season larval containers. This is important because only larger water containers are treated with chemical larvicides in Mexico, whereas community-based cleaning campaigns are supposed to eliminate these “trash” containers. However, the final destiny of trash containers is poorly documented after they are collected and transported by the city waste disposal trucks. It is not difficult to suspect that they are not treated with mosquito egg ovicide actions despite the resilient desiccated eggs glued to their walls. Additionally, mosquitoes, like other insects, have an R reproductive strategy, which means the egg-laying of overwhelming offspring to survive harsh conditions. We argue that quiescent egg populations may reinfest the same areas in the following year when favorable factors such as consistent rains return. Other than increasing reports of dengue vertical transmission elsewhere, the ability of quiescent eggs to undergo either Rapid Hatching (RH) or Delayed Hatching (DH) is an evolutionary trait allowing for great colonizing success of both *Ae. aegypti* and *Ae. albopictus*.

Specific treatments must be targeted in the field to control dry season egg populations. We have shown in this study that spraying dry eggs with Temephos 5% dissolved in mineral oil may be an interesting measure applied during the dry season months to prevent future rainy season outbreaks. Additional studies on the role of egg populations of *Aedes* vectors are urgently needed, considering large outbreaks of dengue, Chikungunya, and Zika continue to be reported in Mexico and Latin America, and vaccines and antiviral drugs will take time to be developed.

## Figures and Tables

**Table 1:** Numbers of adult *Ae. aegypti* and *Ae. albopictus* reared from quiescent eggs collected from inner dry walls of household containers in Tapachula Chiapas, Mexico, 2023.

House #	Dry Egg-Carrying Container	Sample Size	*Ae. aegypti*		*Ae. albopictus*		Total Adults
			Male	Female	Male	Female	
7	toilet tank	1	4	2	13	9	28
15	1 L bottle	1	2	4	4	3	13
43	used tire	1	34	39	24	10	107
64	used tire	1	4	7	7	2	20
100	used tire	1	3	8	3	0	14
122	2 L pan	1	3	3	0	0	6
	total	6	50	63	51	24	188

## Data Availability

Data included within this article.

## References

[1] Pan American Health Organization (OPS) (2024). Handbook for Integrated Vector Management in the Americas. https://iris.paho.org/handle/10665.2/51759.

[2] Fernández-Salas I., Díaz-González E.E., López-Gatell H., Alpuche-Aranda C. (2016). Chikungunya and Zika virus dissemination in the Americas: Different arboviruses reflecting the same spreading routes and poor vector-control policies. Curr. Opin. Infect. Dis..

[3] Ibañez-Bernal S., Briseño B., Mutebi J.P., Argot E., Rodriguez G., Martinez-Campos C., Paz R., La Fuente-San Roman P.D., Tapia-Conyer R., Flisser A. (1997). The first record in America was of *Aedes albopictus* naturally infected with dengue virus during the 1995 outbreak in Reynosa, Mexico. Med. Vet. Entomol..

[4] Clements A.N. (2011). The Biology of Mosquitoes: Viral, Arboviral and Bacterial Pathogens.

[5] Alvarado-Moreno M.S., Laguna-Aguilar M., Rodríguez O.S.S., Sánchez-Casas R.M., Ramírez-Jiménez R., Zarate-Nahón E.A., Achee N., Grieco J.P., Fernández-Salas I. (2013). Potential community-based control by use of plastic film to block *Aedes aegypti* (L.) egg adhesion. Southwest. Entomol..

[6] Danis-Lozano R., Díaz-González E.E., Malo-García I.R., Rodríguez M.H., Ramos-Castañeda J., Juárez-Palma L., Ramos C., López-Ordóñez T., Mosso-González C., Fernández-Salas I. (2019). Vertical transmission of dengue virus in *Aedes aegypti* and its role in the epidemiological persistence of dengue in Central and Southern Mexico. Trop. Med. Int. Health.

[7] Denlinger D.L., Armbruster P.A. (2016). Molecular physiology of mosquito diapause. Advances in Insect Physiology.

[8] (2023). MarketDataMexico. https://www.marketdatamexico.com/es/article/Colonia-Las-Americas-Secc-I-Tapachula-Chiapas.

[9] INEGI (2024). Instituto Nacional de Estadística y Geografía. Información por Entidad. Chiapas. Territorio. Clima. https://cuentame.inegi.org.mx/monografias/informacion/chis/territorio/clima.aspx?tema=me&e=07#:~:text=M%C3%A1s%20de%20la%20mitad%20de,C%20en%20la%20Llanura%20Coste%C3%B1a.

[10] (2023). Boletin de Epidemiologia. Sistema Nacional de Vigilancia Epidemiológica. Secretaría de Salud, Mexico. https://www.gob.mx/cms/uploads/attachment/file/879365/sem52.pdf.

[11] Clark-Gil S., y Darsie R.F. (1983). Los mosquitos de Guatemala. Mosq. Syst..

[12] World Health Organization (WHO) (2008). WHO Specifications and Evaluations for Public Health Pesticides. https://extranet.who.int/prequal/sites/default/files/vcp-documents/WHOVC-SP_Temephos_2010.pdf.

[13] Zar J. (2008). Statistical Analysis.

[14] World Health Organization (2009). Guidelines for Diagnosis, Treatment, Prevention, and Control.

[15] Day J.F. (2016). Mosquito Oviposition Behavior and Vector Control. Insects.

[16] Singh A., Vonk J., Shackelford T. (2019). r-Reproductive Strategy. Encyclopedia of Animal Cognition and Behavior.

[17] Bennett K.L., Gómez Martínez C., Almanza A., Rovira J.R., McMillan W.O., Enriquez V., Barraza E., Diaz M., Sanchez-Galan J.E., Whiteman A. (2019). High infestation of invasive *Aedes* mosquitoes in used tires along the local transport network of Panama. Parasites Vectors.

[18] Morrison A.C., Sihuincha M., Stancil J.D., Zamora E., Astete H., Olson J.G., Vidal-Ore C., Scott T.W. (2006). Producción de *Aedes aegypti* (Diptera: Culicidae) en sitios no residenciales de la ciudad amazónica de Iquitos, Perú. Ann. Trop. Med. Parasitol..

[19] World Health Organization (2017). Global Vector Control Response 2017-20130. CC BY-NC-SA 3.0 IGO. http://apps.who.int/iris.

